# Items from a Case Book

**Published:** 1860-11

**Authors:** H. D. Capers

**Affiliations:** Atlanta, Georgia


*Items from a Case Book.* By H. D. CAPERS, M. D., Atlanta, Georgia.

*Mr. Editor:* Below I present the history of a case taken from my case-book, which, allow me to hope, will prove of some interest to the many readers of your valuable Journal. The little progress made in the management of Dropsical affections, and the earnest desire to contribute something towards the amelioration of the sufferings to which our race is heir, determined me to do so.

I visited on the 10th of May, 1859, Mrs. F., who had recently been conducted through the anguish of a protracted labor by one of woman's curses—an old, senseless "Granny," with more prejudice than brains, and more conceit than either. I found the sufferer a lady of delicate frame, and, from her complexion and general anœmic appearance, of a dropsical diathesis. Her respiration was hurried, and quite audible across the chamber—the slightest exertion producing a most distressing paroxysm of dyspnœa. Pulse very rapid and compressible; tongue slightly coated; skin very dry, cool and puffy; bowels constipated; lochial discharge ceased entirely; in fact, every unmistakable symptom of Hydrothorax. Upon auscultating the thorax, the respiratory murmur was scarcely audible, and the absence of all resonance was detected by percussion. The stetescope revealed the contractions of the heart very indistinct and frequently intermitting. Mrs. F. had noticed a slight enlargement of her feet and hands towards the close of her term, and first became aware of her thoracic affection during her accouchment which was protracted through the tedium and sufferings of three weary days. I pronounced the case Hydrothorax, and from the excessive debility and delicate constitution of my patient, gave no very flattering prognosis. I ordered for the evening

Pul. Jallapa, grs. x,
B: Tar. Potassa, grs. v.

To be given every three hours, with Sul. Morphia grs. $\frac{1}{4}$ intermediate.

May 11.—Mrs. F. spent a more agreeable night than usual, thanks to the opiate. Pulse still very quick and feeble, 154 beats in a moment; tongue furred; respiration very hurried, with great oppression; no respiratory murmur, and the sounds of the heart very dull. The cathartic had produced but a slight evacuation, and I resolved to repeat it in connection with the following:

Tinct. Digitalis, ʒ i.,

Tinct. Cimicifuga, ℥ ss.

Tea-spoonful every three hours.

May 12th.—My patient spent a very unpleasant evening, constantly troubled with distressing paroxysms of dyspnœa—find no alteration in the pulse either as to volume or force. Respiration exceedingly rapid—up to this time the excessive debility of Mrs. F. forbid the use of the Lancet; the Digitalis last evening had scarcely any effect, either upon the heart or kidneys. I resolved, therefore, to make an attempt at controlling the heart's action with Dr. Norwood's tincture of the American Hellebore, believing that its specific effects would produce a material change in her condition. I gave it in four minim doses at intervals of three hours and repeated the cathartic.

At two o'clock P. M., I was summoned in great haste to my patient—found her vomiting, as she had been since the second dose of the Veratria—pulse reduced to 72; profuse diaphoresis—immediately administered Norwood's antidote, with but little effect. An encema of two drachms McMunn's Elixir of Opium arrested the emesis promptly.

It was really gratifying to witness the great change that had so speedily taken place in the sufferer's condition. The family believed her very near death, and my old "Granny" friend became greatly displeased when I pronounced Mrs. F. better than at any time since my first visit. The powerful sedative effect of the Tincture had wrought wonders in her case: the distressing dyspnœa had ceased entirely, a profuse diaphoresis had been established, and the kidneys excited into a happy discharge of their function. Late in the evening she was sweetly asleep, her pulse at no time rising above 87 beats to the minute. Just before morning the cathartic produced a copious evacuation, and I was happy in observing a return of the lochial discharge, which, as before remarked, had entirely ceased.

I considered the battle fought, and next morning ordered but two minim doses of the Veratria, at intervals of three hours, to keep the heart's action under control, and my patient to drink freely of a tea prepared from the ashes of the wild grape vine, a most convenient mode of administering a valuble diuretic.

Afternoon—Mrs. F's. condition greatly improved—action of the thorax normal—vesicular murmur quite audible, auricular and ventricular contractions of the heart quite distinct—pulse 84 with a decided increase in volume—kidneys acting well and the lochia quite returned—ordered an opiate for the evening and the use of the grape tea as a substitute for water.

May 15th.—My patient meets me this morning with that meaning smile, so well known and highly appreciated by the attending physician. Her respiration is quite natural and but little evidence of any existing effusion. I decline the further use of the sedative, and order for the day, the exhibition of a gentle catharic and the continuance of the potassa.

May 16th.—Pulse exhibits no tendency to rise, continuing at from 84 to 86, without the use of Dr. Norwood's Tinture Lochia entirely restored—skin pleasant and tongue cleaned off—ordered for Mrs. F. Quevenne's preparation of iron in pills of two grains each, and a small glass of wine at midday.

August 12.—Had the pleasure of an interview with Mrs. F.—found her actively engaged in the discharge of her domestic duties—entirely free from any thoracic or general dropsical effusion, and I may add up to September of this year, when I lost sight of her, in the enjoyment of excellent health.

Remarks upon the above detailed case referred to the next number of the Journal.

